# Biosynthesis of Antituberculosis Antibiotic Capreomycin Involves a *trans*-Iterative Adenylation Domain within the Nonribosomal Peptide Synthetase Machinery

**DOI:** 10.1021/acs.orglett.5c03112

**Published:** 2025-08-15

**Authors:** Yi-Ting Lai, Chun-Yu Peng, Hsiao-Tzu Liao, Po-Yun Hsiao, Chang-Ko Hsieh, Yu-Ru Luo, Sheng-Cih Huang, Yung-Lin Wang, Thomas Ma, Ya-Rong Chen, You-Min Kuo, Yen-Cheng Lin, John Chu, Chin-Yuan Chang

**Affiliations:** ‡ Department of Biological Science and Technology, 34914 National Yang Ming Chiao Tung University, Hsinchu 30010, Taiwan; § Department of Applied Chemistry, National Yang Ming Chiao Tung University, Hsinchu 30010, Taiwan; ∥ Genomics Research Center, 38017 Academia Sinica, Taipei 11529, Taiwan; ⊥ Department of Chemistry, 33561 National Taiwan University, Taipei 10617, Taiwan; # Center for Intelligent Drug Systems and Smart Biodevices (IDS2B), National Yang Ming Chiao Tung University, Hsinchu 30010, Taiwan; ∇ Center for Emergent Functional Matter Science, National Yang Ming Chiao Tung University, Hsinchu 30010, Taiwan; ○ Department of Biomedical Science and Environmental Biology, Drug Development and Value Creation Research Center, Kaohsiung Medical University, Kaohsiung 807, Taiwan

## Abstract

Capreomycin (CMN) is a nonribosomal peptide (NRP) antituberculosis antibiotic. CMN biosynthesis involves a non-canonical *trans*-iterative adenylation (A) domain. Here, we report that the A domain-less nonribosomal peptide synthetase (NRPS) module CmnI utilizes another module’s A domain CmnA-A_1_ to load the required amino acid onto its thiolation (T) domain. This study provides evidence of an unusual mode of NRP biosynthesis in bacteria, involving a *trans*-iterative A domain in the NRPS machinery.

## Graphical abstract

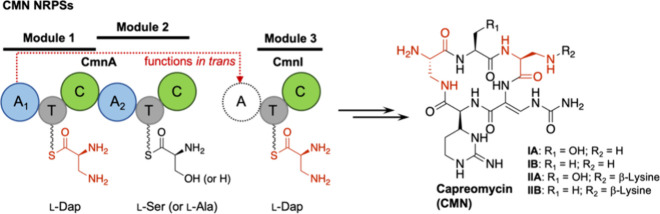

Nonribosomal peptides (NRPs) represent a major class of natural products, mainly produced by bacteria and fungi with clinical examples, including the antibiotic vancomycin,^1^ anticancer bleomycin,^2^ and immunosuppressant cyclosporins.^3^ Their biosynthesis is mediated by nonribosomal peptide synthetases (NRPSs), a family of multidomain megaenzymes that catalyze peptide bond formation and assemble the peptide chain.^4^ Typically, each NRPS module consists of three core domains, an adenylation (A) domain, a condensation (C) domain, and a thiolation (T) domain [also known as a peptidyl carrier protein (PCP) domain].^5^ However, nature has evolved diverse NRPS machineries, including an iterative system found in bacterial natural products enterobactin,^6^ gramicidin,^7^ solanimycin,^8^ syrinogomycin,^9^ and yersinabactin,^10^ as well as in fungal natural products beauvericin,^11^ bassianolide,^12^ and verticilide.^13^ Interestingly, in fungal siderophore biosynthesis, some NRPSs, such as SidD, Nps6, and SidC, lack their own A domain and rely on upstream A domains for amino acid activation and loading onto their T domains.^14^

Capreomycin (CMN) and viomycin (VIO) are tuberactinomycin antibiotics produced by *Saccharothrix mutabilis* subsp. *capreolus* and *Streptomyces* sp. strain ATCC 11861, respectively (Figure 1a).^15^ Their biosynthetic gene clusters were identified, and the biosynthetic pathways have also been proposed.^16^ The cyclic pentapeptide cores of CMN and VIO are synthesized by five NRPS modules encoded in four enzymes, CmnA/VioA, CmnI/VioI, CmnF/VioF, and CmnG/VioG (Figure 1b). Intriguingly, CmnI and VioI in module 3 lack their own A domain and must rely on another module for substrate activation. Previous studies suggested that the first module A domain of CmnA (CmnA-A_1_) in CMN biosynthesis and the second module A domain of VioA (VioA-A_2_) in VIO biosynthesis aminoacylate the T domain of CmnI and VioI, respectively (Figure 1b).^16^ Here, we report a crosstalk between two NRPS modules in CMN biosynthesis, where CmnA-A_1_ functions in *trans*, loading l-2,3-diaminopropionic acid (l-Dap) to CmnI. Similar to fusarinine C biosynthesis in a fungus,^14^ a comparable mechanism also exists in bacteria, where a single A domain activates and loads the substrate onto two T domains across separate NRPS modules. The discovery of such crosstalk expands our understanding of NRPS machinery in bacteria and provides insights into the flexibility in NRP biosynthesis.

**Figure 1:**
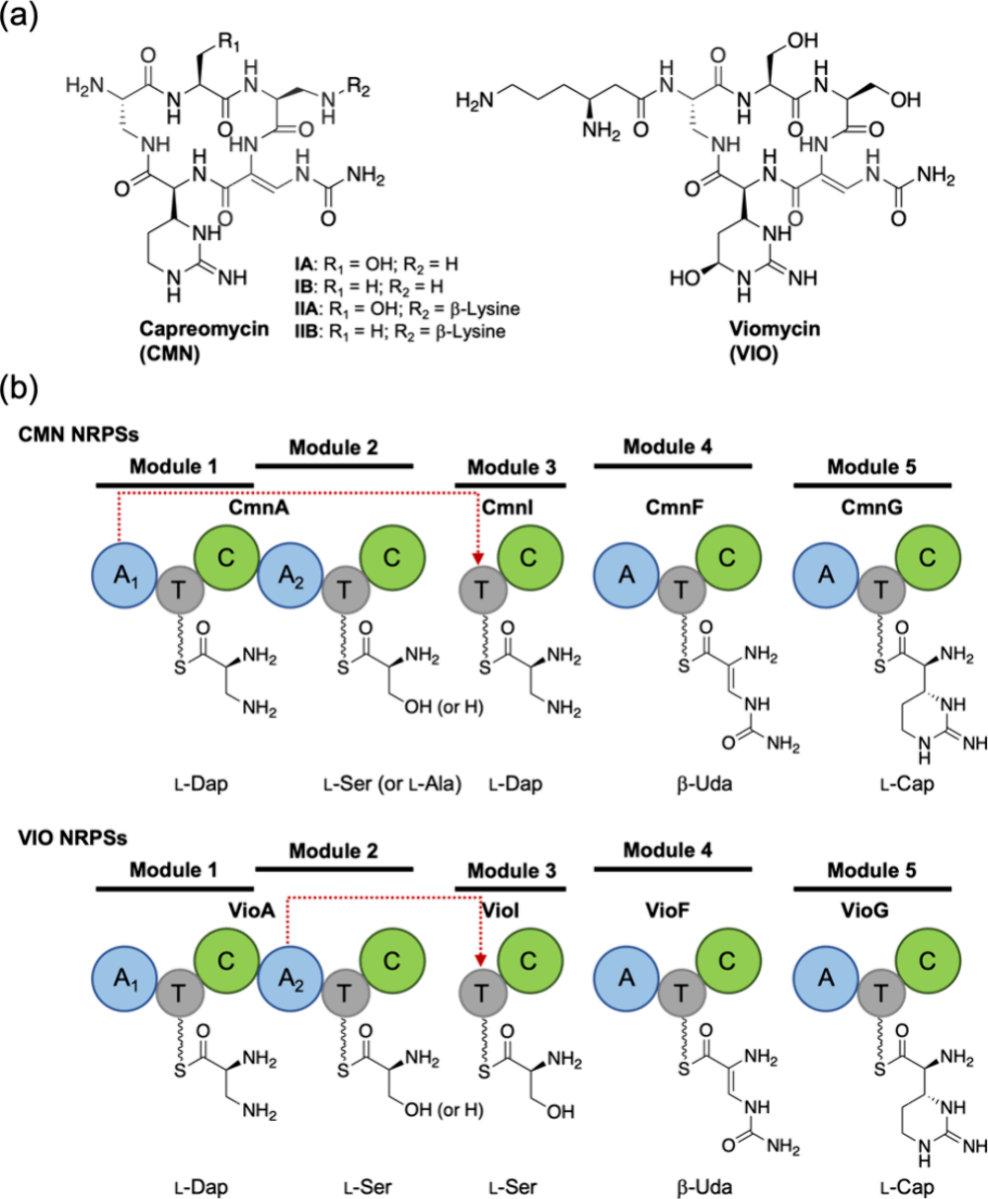
Biosynthesis of CMN and VIO: (a) Structures of the four CMN derivatives and VIO and (b) composition and arrangement of the NRPSs of CMN and VIO. In both CMN and VIO, NRPS module 3 lacks an A domain. l-Cap and β-Uda represent l-capreomycidine and β-ureidodehydroalanine, respectively. The red dashed arrows indicate that CmnA-A_1_ and VioA-A_2_ load amino acids across modules onto CmnI-T and VioI-T, respectively.

Previous studies have shown that the MbtH-like protein CmnN, found within the CMN biosynthetic gene cluster, binds to CmnA-A_1_ and plays a crucial role in stabilizing the A domain structure to facilitate its enzyme activity.^17^ We further demonstrated that CmnA-A_1_ specifically recognizes and activates the nonproteinogenic amino acid l-Dap, while structurally similar l-Ser and l-Cys as well as l-Ala and l-Lys cannot serve as its substrates (Figures S1 and S2a). In order to demonstrate that CmnA-A_1_ loads l-Dap onto the T domain of CmnI in *trans*, we constructed and transformed *Escherichia coli* BL21­(DE3) with three plasmids: pACYCDuet carrying *cmnA-A*_*1*_ and *cmnN*, pET28a carrying *cmnI*, and pCDFDuet carrying *svp* (phosphopantetheinyl transferase, making CmnI the holo form).^18^ Only *cmnI* possesses a N-terminal His-tag. After gene expression, CmnI was purified using Ni-NTA (Figure S1), and its molecular weight was subsequently analyzed by using Q-TOF mass spectrometry. With expression of only *cmnI* in *E. coli*, the T domain of CmnI is not phosphopantetheinylated and the molecular mass represents apo-form CmnI. With expression of *cmnI* and *svp* in *E. coli*, CmnI became the holo form with a shift of +340 Da compared to that of apo-form CmnI (Figure 2a and 2b), indicating phosphopantetheinylation of the T domain. With additional expression of *cmnA-A*_*1*_ and *cmnN* in *E. coli*, the molecular mass of CmnI revealed additional 86 Da compared to that of holo-form CmnI (Figure 2b and c), indicating l-Dap tethered on the T domain of CmnI. We proposed that l-Dap is naturally present in the intracellular environment of *E. coli*,^19^ allowing CmnA-A_1_ to load l-Dap onto the T domain of CmnI without the need for externally added l-Dap. When we added l-Dap to the culture of the *E. coli* construct expressing *cmnA-A*_*1*_, *cmnN*, *cmnI*, and *svp*, the signal of the molecular mass of CmnI loaded with l-Dap was relatively enhanced (Figure 2c and d). This result confirmed that the A domain in module 1 is capable of iterative action and in *trans* loading l-Dap onto CmnI’s T domain within module 3.

**Figure 2:**
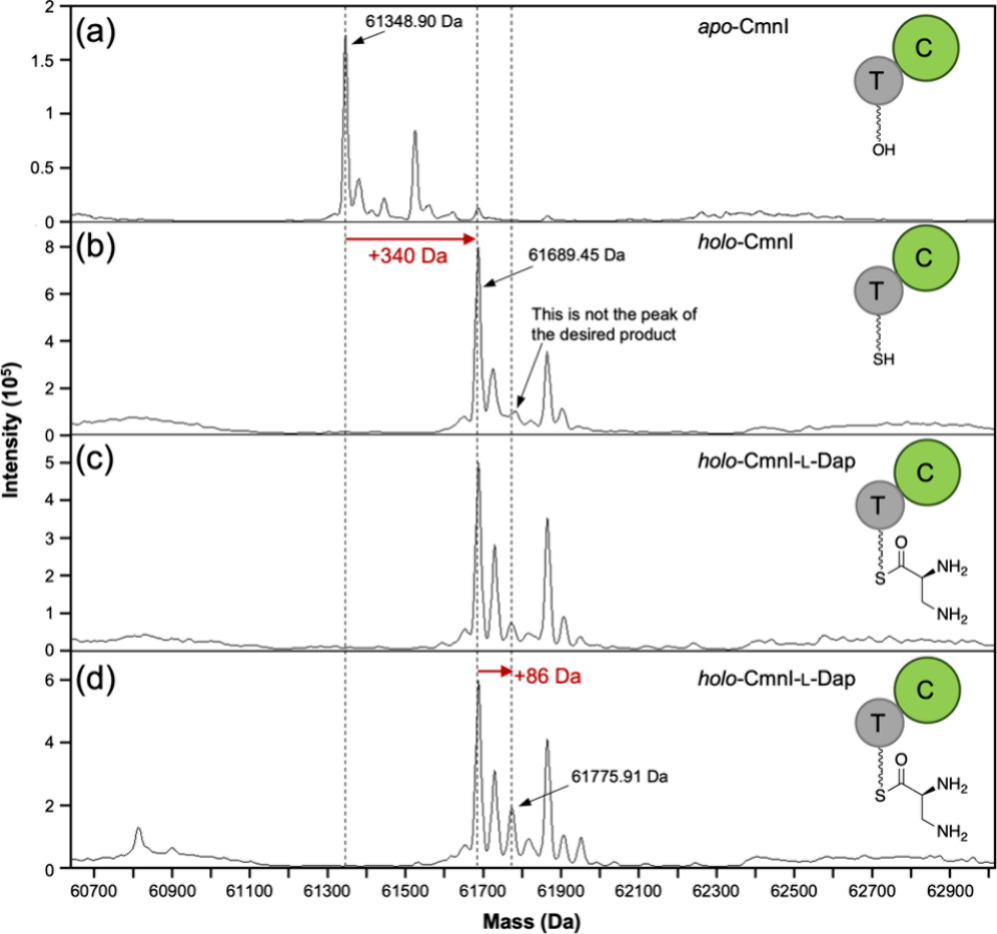
*In vivo* enzymatic reactions and mass analysis of CmnI. Deconvoluted protein mass spectra of purified CmnI: (a) apo-form CmnI (expression of only *cmnI* in *E. coli*), (b) holo-form CmnI (expression of *cmnI* and *svp* in *E. coli*), and (c and d) CmnI loaded with l-Dap (co-expression of *cmnI*, *svp*, *cmnA-A*_*1*_, and *cmnN* in *E. coli*). Spectra c and d represent the conditions without and with l-Dap added to the culture medium, respectively. The calculated molecular weight of apo-form CmnI is 61 484.72 Da.

We then conducted a similar experiment to determine whether the T domain of CmnI (CmnI-T) can be loaded with l-Dap by CmnA-A_1_ in the absence of its C-terminal C domain. However, the expression level of *cmnI-T* was low when co-expressed with *cmnA-A*_*1*_, *cmnN*, and *svp*. As a result, we were unable to obtain a sufficient amount of CmnI-T for mass analysis. Consequently, we performed an experiment in which we purified CmnI-T and the CmnA-A_1_–CmnN complex separately (Figure S1) and then mixed them to observe whether l-Dap can be loaded onto CmnI-T. The substrate l-Dap, along with the cofactors ATP and Mg^2+^, were included in the enzyme reaction. Following *in vitro* reaction, Q-TOF analysis of CmnI-T revealed an additional 86 Da compared to the holo form of CmnI-T (Figure 3), indicating that CmnA-A_1_ is responsible for loading l-Dap onto CmnI-T. This result shows that CmnA-A_1_ is functional even without the C domain.

**Figure 3:**
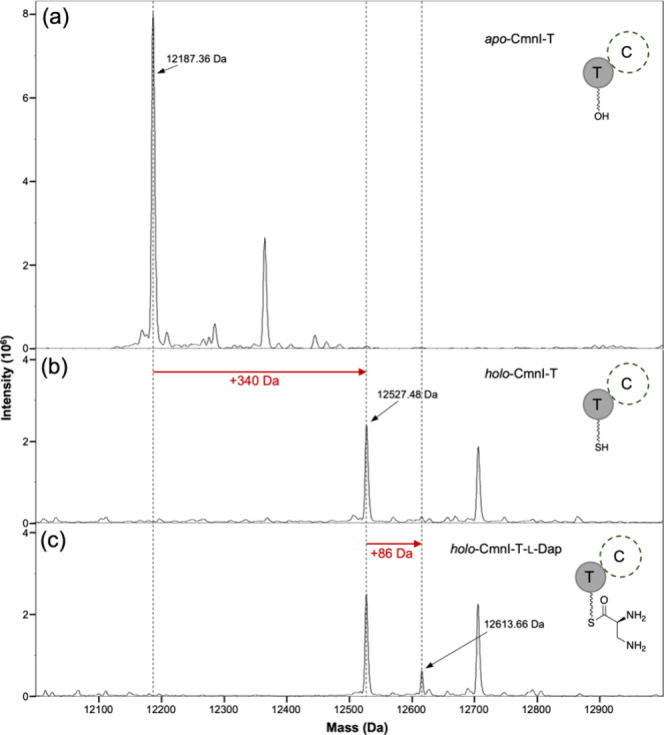
*In vitro* enzymatic reactions and mass analysis of CmnI-T: (a) apo-form CmnI-T, (b) holo-form CmnI-T, and (c) CmnI-T loaded with l-Dap. In the reaction of spectrum c, CmnI-T, CmnA-A_1_–CmnN, as well as the substrate l-Dap and the cofactors ATP and Mg^2+^ were added. Subsequently, CmnI-T was purified for an intact protein mass analysis. The calculated molecular weight of apo-form CmnI-T is 12 316.88 Da.

To investigate the interaction between CmnA-A_1_–CmnN and holo-form CmnI, size-exclusion chromatography was performed to assess changes in protein size. The result showed no detectable change compared to CmnA-A_1_–CmnN and holo-form CmnI individually (Figure S3), suggesting a lack of strong interaction between the two proteins under the tested conditions. We then utilized biolayer interferometry (BLI) to further examine whether CmnA-A_1_–CmnN interacts with CmnI. The measured dissociation constant (*K*_D_) was 1.85 μM (*K*_on_ = 258 M^–1^ s^–1^ and *K*_off_ = 4.77 × 10–4 s^–1^) (Figure S4a), indicating a weak binding affinity between CmnA-A_1_–CmnN and CmnI. Furthermore, we expressed, produced, and purified CmnA-A_2_ for the enzyme activity assay. CmnA-A_2_ is capable of activating both l-Ser and l-Ala and also interacts with CmnN to stabilize its function (Figures S1 and S2b). In contrast, measured *K*_D_ between CmnA-A_2_-CmnN and CmnI was 4.93 μM (*K*_on_ = 148 M^–1^ s^–1^ and *K*_off_ = 7.28 × 10–4 s^–1^) (Figure S4b), which is weaker than that of CmnA-A_1_–CmnN and CmnI. These results suggest that CmnI has a stronger binding preference for CmnA-A1 compared to CmnA-A_2_, which may account for the efficient *trans*-loading of l-Dap onto the T domain of CmnI. These results provide evidence of crosstalk between CmnA-A_1_–CmnN and CmnI, supporting the dynamics of the NRPS machinery, where CmnA-A_1_ functions iteratively, acts on the T domain of module 1, and then engages with that of module 3 in *trans*.

Although increasing numbers of NRPS modules lacking an A domain have been identified, experimental validation of *trans*-acting A domain mechanisms in bacteria remains limited. This study provides experimental evidence that the A domain from one module loads the substrate onto a separate A domain-less module, demonstrating *trans*-activation in the bacterial system. To explore the distribution and evolution of such modules, we performed a BLASTP search against GenBank and retrieved over 1000 CmnI homologues (30–100% identity), all annotated as NRPSs. We constructed a sequence similarity network (SSN) using 987 homologues of CmnI retrieved from the UniProt database with sequence identities greater than 35% with CmnI at an *e* value threshold of 1 × 10^–80^. The resulting SSN revealed a distinct group consisting of 13 CmnI homologues, including CmnI and VioI (Figure 4a). These clustered proteins share 52.5–99.5% identity, and all lack an A domain (Figures S5 and S6). Gene cluster analysis via antiSMASH^20^ confirmed that all 13 CmnI or its homologues are associated with CMN/VIO biosynthetic gene clusters (Figure 4b). These results suggest that CmnI represents a distinct class of NRPS modules, and the SSN provides a bioinformatics framework to identify potential producers of this antibiotic family.

**Figure 4:**
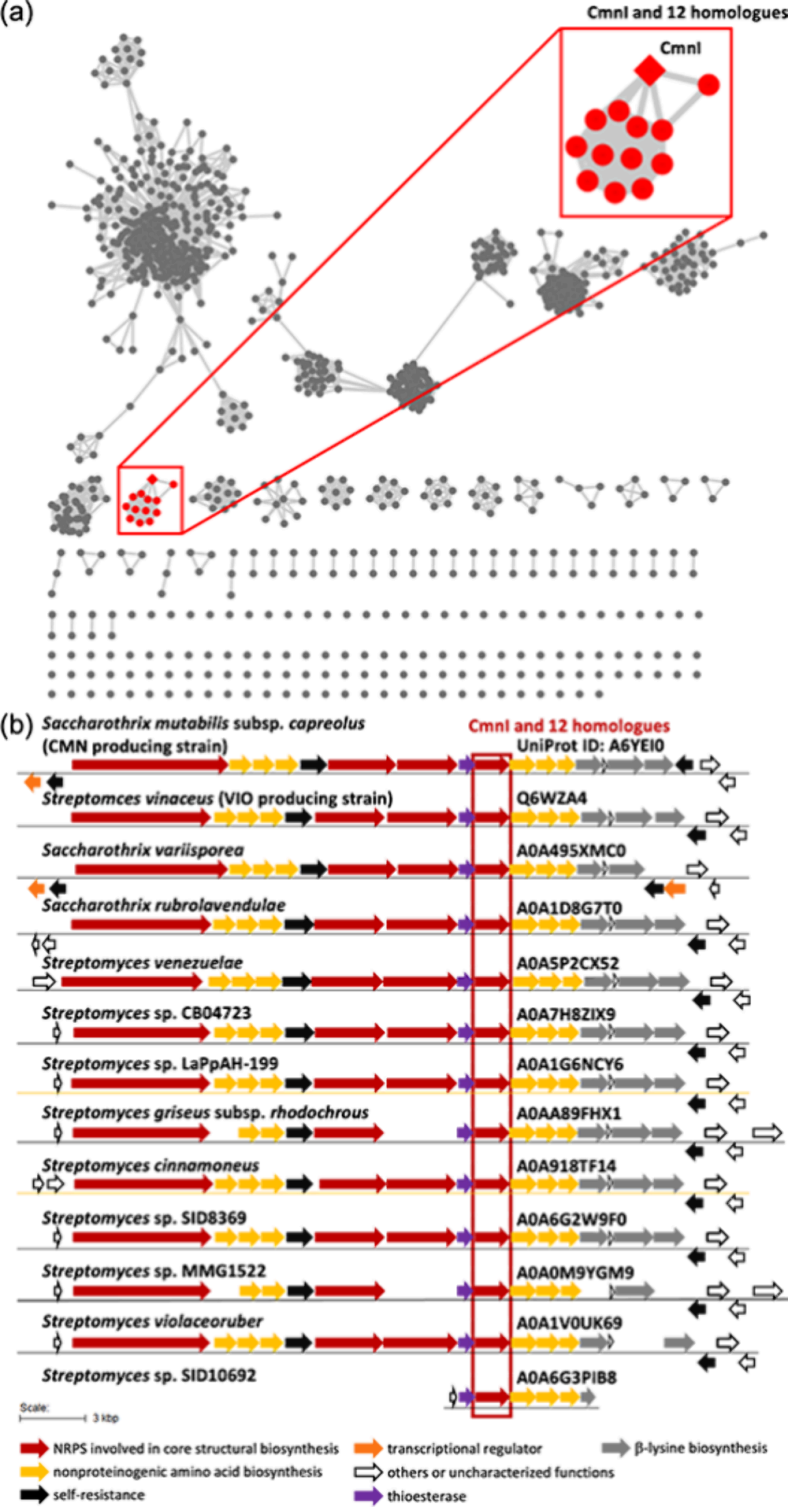
Bioinformatics analysis of the CmnI homologues: (a) 987 CmnI homologues classified by the SSN analysis at an *e* value of 10^–80^, with CmnI and the 12 homologues forming a distinct group in the SSN, highlighted in red, and (b) biosynthetic gene clusters identified by antiSMASH containing 12 CmnI homologues highlighted in panel a. The proposed biosynthetic functions of the open reading frames (ORFs) are indicated below the figure.

To investigate whether CmnI possesses unique structural characteristics, X-ray crystallography was carried out to determine the structure of CmnI. The crystal structure of CmnI was determined using the molecular replacement (MR) method, with the structure of the T–C didomain NRPS from fuscachelin biosynthesis (PDB entry 7KVW)^21^ as the initial search model. The final model of CmnI was refined to a resolution of 2.17 Å (Table S1). However, we observed only the C domain, while the structure of its T domain could not be determined, likely due to its flexibility. CmnI was crystallized as a monomer, consistent with size-exclusion chromatography (Figure S7). The C domain of CmnI (CmnI-C) shares a similar three-dimensional structure with canonical C domains.^22^ The overall structure of CmnI-C is mainly divided into two parts, N-terminal and C-terminal subdomains (Figure 5a). The floor loop (Val387–Asn404) and the bridging region (Ala464–Phe494) contact two subdomains, forming a continuous tunnel spanning the entire C domain. The PEG molecules are located in the tunnel, suggesting the entry pathway for peptide and amino acid substrates. As with canonical C domains, the conserved HHXXXD motif (His248–Asp253) is located at the center of this tunnel, suggesting its essential role in catalysis (Figure 5b). However, despite the structural similarities between CmnI-C and typical C domains, SSN analysis grouped 13 CmnI homologues into a distinct group (Figure 4a), indicating significant sequence divergence. This suggests that, while CmnI retains the canonical fold, its sequence variations may confer functional specificity. The T domain of CmnI is around 20 residues longer than typical T domains (∼80 amino acids).^22^ Additionally, almost all CmnI homologues share this extended N-terminal region (Figure S5). AlphaFold3 modeling^23^ predicts this extension at the N terminus of CmnI to be flexible and disordered (Figure 5c). This N-terminal region of CmnI might be involved in the communication with the CmnA-A_1_ domain. We next expressed and purified a N-terminally truncated variant of CmnI (CmnI_21–548_) (Figure S1), lacking the first 20 residues, and determined the binding ability to CmnA-A_1_ using BLI. During the assay, CmnI_21–548_ was gradually precipitated over time upon mixing with CmnA-A_1_, suggesting that removal of the N-terminal region compromises the stability of the interaction between CmnI and CmnA-A_1_. The BLI results showed no detectable protein–protein interaction (Figure S4c). This finding supports the hypothesis that the extended N-terminal region of CmnI plays an important role in mediating communications with CmnA-A_1_ and stabilizing the protein–protein interaction.

**Figure 5:**
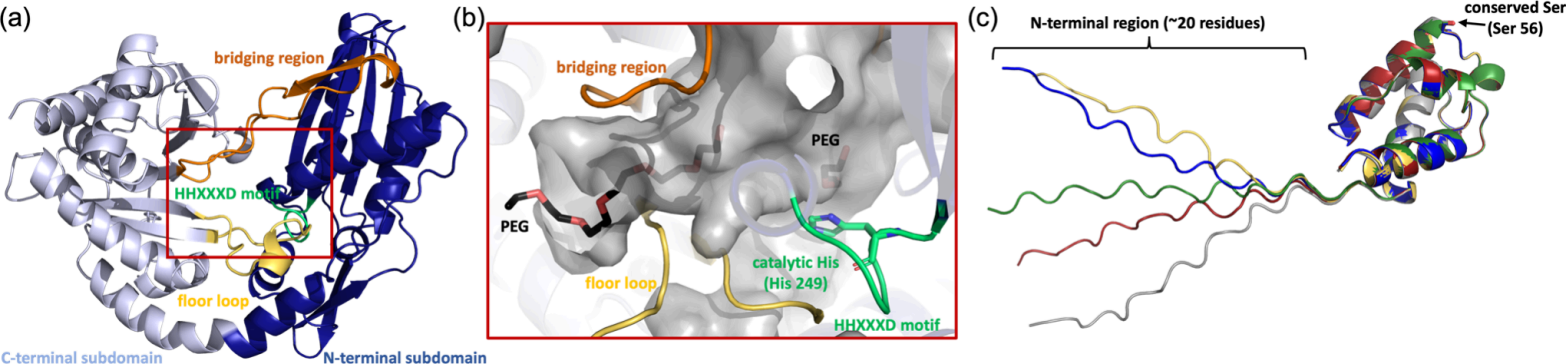
Structure of CmnI: (a) crystal structure of the C domain of CmnI (CmnI-C), with the N- and C-terminal subdomains colored deep blue and light blue, respectively, the floor loop and bridging region colored yellow and orange, respectively, and the C domain conserved motif HHXXXD colored green, (b) local view of the putative CmnI-C active-site cavity, with the second histidine, His249, of the conserved motif shown as green sticks and PEG shown as black sticks, and (c) AlphaFold models of the T domain of CmnI (CmnI-T), with the N-terminal region of CmnI-T predicted to be a disorder region, the five predicted models of CmnI-T shown in five different colors, and the conserved T domain serine, Ser56, shown as sticks.

To investigate the potential interactions between CmnA-A_1_ and the two T domains, we generated two complex models, CmnA-A_1_–CmnN–CmnA-T_1_ and CmnA-A_1_–CmnN–CmnI-T, using AlphaFold3. Both models exhibit high structural similarity to the crystal structure of a NRPS EntF (PDB entry 5JA1),^24^ in which the A domain, T domain, and MbtH-like protein are located in comparable positions and orientations (Figure S8a). This resemblance supports the validity of our predicted models. In both the CmnA-A_1_–CmnN–CmnA-T_1_ and CmnA-A_1_–CmnN–CmnI-T models, CmnA-A_1_ forms an electrostatic interaction via Glu611 with a conserved arginine residue, Arg932 of CmnA-T_1_ or Arg61 of CmnI-T (Figure S8b and c). In addition, CmnA-T_1_ forms hydrogen-bond interactions with CmnA-A_1_ through the side-chain hydroxyl groups of Thr931 and Ser927, which interact with the backbone carbonyl group and side-chain hydroxyl group of Thr582 in CmnA-A_1_, respectively (Figure S8b). In the CmnI-T complex, Arg65 and Arg68 form electrostatic interactions with Glu612 and Asp614 of CmnA-A_1_, respectively (Figure S8c). The structural models support the BLI data, suggesting a direct interaction between CmnI and CmnA-A_1_.

In conclusion, unlike typical NRPS modules, CmnI lacks its own A domain. CmnI relies on the *trans*-iterative A domain, CmnA-A_1_, from another NRPS module to load l-Dap into its T domain. BLI analysis confirmed that CmnI and CmnA-A_1_ interact with micromolar affinity, supporting functional cross-talk between these two modules. Structural analysis revealed that CmnI closely resembles canonical C domains; however, the T domain of CmnI features an extended N-terminal region compared to typical T domains. SSN analysis revealed that 13 CmnI homologues are classified into a distinct group, which helps guide the search for additional CMN/VIO-type antibiotics. This study provides new insights into NRPS machinery in bacteria, expanding our understanding of their mechanisms and potential applications in natural product engineering.

## Supplementary Material



## Data Availability

The data underlying this study are available in the published article and its Supporting Information.

## Notes

Experimental details including gene cloning and expression, protein purification and crystallization, mass spectrometric analysis, structural determination, enzyme activity assay, BLI binding kinetic assay, and SSN analysis (PDF)
