# Supplementary material for: Biosynthesis of Antituberculosis Antibiotic Capreomycin Involves a trans-Iterative Adenylation Domain within the Nonribosomal Peptide Synthetase Machinery
Source: Org Lett. 2025 Aug 15;27(34):9553–8. doi: 10.1021/acs.orglett.5c03112 (PMC12400406; doi:10.1021/acs.orglett.5c03112)
Supplement: Supplementary file 1 [file ol5c03112_si_001.pdf]

Supporting Information

Biosynthesis of antituberculosis antibiotic capreomycin involves a *trans*-iterative adenylation domain within the NRPS machinery

Yi-Ting Lai,[+,1] Chun-Yu Peng,[+,1] Hsiao-Tzu Liao,[1] Po-Yun Hsiao,[1] Chang-Ko Hsieh,[1] Yu-Ru Luo,[1] Sheng-Cih Huang,[2] Yung-Lin Wang,[3] Thomas Ma,[4] Ya-Rong Chen,[4] You-Min Kuo,[1] Yen-Cheng Lin,[1] John Chu,[4] and Chin-Yuan Chang[*,1,5,6,7]

[1]Department of Biological Science and Technology, National Yang Ming Chiao Tung University, Hsinchu 30010, Taiwan

[2]Department of Applied Chemistry, National Yang Ming Chiao Tung University, Hsinchu 30010, Taiwan

[3]Genomics Research Center, Academia Sinica, Taipei 11529, Taiwan

[4]Department of Chemistry, National Taiwan University, Taipei 10617, Taiwan

[5]Center for Intelligent Drug Systems and Smart Biodevices (IDS^{2}B), National Yang Ming Chiao Tung University, Hsinchu 30010, Taiwan

[6]Center for Emergent Functional Matter Science, National Yang Ming Chiao Tung University, Hsinchu 30010, Taiwan

[7]Department of Biomedical Science and Environmental Biology, Drug Development and Value * Creation Research Center, Kaohsiung Medical University, Kaohsiung 807, Taiwan

+These authors contributed equally to this work

*** Corresponding author:**

Dr. Chin-Yuan Chang, E-mail: cycytl@nycu.edu.tw

Table of Contents

Supplementary Methods

Gene cloning, protein production and purification of the CmnA-A_1-CmnN complex and the CmnA-A_2-CmnN complex……………………………………………………………………………………S3

Gene cloning, protein production and purification of CmnI, CmnI-T, and $CmnI_{21-548}$……………………S3

Activity assay of the CmnA-A_1-CmnN complex and the CmnA-A_2-CmnN complex……………………….S4

Mass spectrometric analysis of apo-form CmnI/CmnI-T, holo-form CmnI/CmnI-T, and CmnI/CmnI-T loaded with L-Dap……………………………………………………………………………………S4

Determination of the size of CmnI by size-exclusion chromatography……………………………S4

Bio-layer interferometry (BLI) binding kinetics assay………………………………………………S5

Crystallization and data collection of CmnI……………………………………………………………S5

Structure determination and refinement of CmnI………………………………………………………S5

AlphaFold structure prediction……………………………………………………………………………S5

Sequence similarity network (SSN) of CmnI……………………………………………………………S5

Supplementary Table

Table S1. Data collection, phasing, and refinement statistics for CmnI structure…………………………S7

Supplementary Figures

Figure S1. SDS-PAGE and molecular weight estimation of CmnI, CmnI-T, $CmnI_{21-548}$, the CmnA-A_1-CmnN complex, and the CmnA-A_2-CmnN complex……………………………………………………….S8

Figure S2. Substrate selectivity of CmnA-A_1……………………………………………………..S9

Figure S3. Size-exclusion chromatography analysis of interaction between CmnA-A_1 and CmnI……....S10

Figure S4. Kinetic binding curves of the CmnA-A_1-CmnN complex and the CmnA-A_2-CmnN complex variants interacting with the holo-form CmnI or $CmnI_{21-548}$…………………………………………...S11

Figure S5. Sequence alignment of CmnI with 12 homologues from the specific cluster……………...S12

Figure S6. Sequence alignment identity matrix of CmnI with 12 homologues from the specific cluster….S13

Figure S7. Molecular weight estimation of CmnI……………………………………………………..S14

Figure S8. Potential interactions between CmnA-A1 and the two T domains…………………………..S15

Supplementary References…………………………………………………………………………S16

Supplementary Methods

Gene cloning, protein production and purification of the CmnA-A$_1$-CmnN complex and the CmnA-A$_2$-CmnN complex

The *cmnN* and *cmnA-A$_1$* (encoding residues Met−His364−Leu851 of CmnA) genes from *Streptomyces mutabilis* subsp. *Capreolus* (ATCC 23892) were synthesized artificially and cloned in expression vector pACYCDuet-1 at MCS-1 (between BamHI and HindIII) and MCS-2 (between NdeI and XhoI), respectively, to generate pACYCDuet-CmnA-A$_1$-CmnN. This construct produces the CmnA-A$_1$-CmnN complex with an N-terminal His-tag on the N-terminus of CmnN. In addition, the *cmnN* gene cloned between NcoI and HindIII produces the CmnA-A$_1$-CmnN complex without His-tag. All plasmids in this study were used to transform *E. coli* BL21 (DE3) for gene expression and protein production. A general procedure of gene expression, protein production and purification are described as follows: 2 L of LB medium was inoculated with 5 mL of an overnight *E. coli* BL21 (DE3) culture grown in LB medium containing 25 μg/mL chloramphenicol, induced with 1 mM isopropyl-β-D-1-thiogalactopyranodie (IPTG) at an OD_{600} of 0.6. Cells were grown for a further 16 hr at 18 ºC and were harvested by centrifugation at 6,000 xg for 30 min at 4 ºC. The cells after gene expression and harvested by centrifugation were resuspended in lysis buffer (500 mM NaCl and 20 mM Tris, pH 8.0). The cells were disrupted by sonication and were then centrifugated at 15,0 00 xg for 30 min at 4 ºC to remove cell debris. The CmnA-A$_1$-CmnN complex was purified using Ni-NTA affinity [HisTrap FF (Cytiva)] and size-exclusion chromatography [HiLoad Superdex 16/600 200 pg (Cytiva)]. All chromatography were performed by NGC Chromatography Systems (Bio-Rad). The N-terminal His-tag was cleaved by thrombin following standard procedures. The protein purity was verified by SDS-PAGE. The purified protein was concentrated using Amicon Ultra-15 10,000 NMWL concentrators (Merck) in 100 mM NaCl and 20 mM Tris buffer at pH 8.0 for the following activity assay, binding assay, and BLI analysis. Protein concentration was determined using the BCA kit (Thermo Scientific) following the standard protocol. The purified protein was stored at −80 ºC.

For the CmnA-A$_2$-CmnN complex, the *cmnN* and *cmnA-A$_2$* (encoding residues Met−Leu1832−Gly1896 of CmnA) genes from *Streptomyces mutabilis* subsp. *Capreolus* (ATCC 23892) were synthesized artificially and cloned in expression vector pACYCDuet-1 at MCS-1 (between BamHI and HindIII) and MCS-2 (between NdeI and XhoI), respectively, to generate pACYCDuet-CmnA-A$_2$-CmnN. The expression, production, and purification of the CmnA-A$_2$-CmnN complex followed the same procedures as those used for the CmnA-A$_1$-CmnN complex.

Gene cloning, protein production and purification of CmnI, CmnI-T, and CmnI$_{21\text{-}548}$

The *cmnI* and *cmnI$_{21\text{-}548}$* genes from *Streptomyces mutabilis* subsp. *Capreolus* (ATCC 23892) were synthesized artificially and cloned in expression vector pET28a (between NdeI and HindIII) to generate pET28a-CmnI and pET28a-CmnI$_{21\text{-}548}$. We then introduced a stop codon after the Gly100 position through mutagenesis to generate pET28a-CmnI-T from pET28a-CmnI. The *svp* gene from *Streptomyces verticillus* (ATCC 15003)[1] was synthesized artificially and cloned in expression vector pCDFDuet-1 at MCS-2 (between NdeI and XhoI)

to generate pCDFDuet-Svp. Without co-expression of *svp*, apo-form CmnI/CmnI-T/$CmnI_{21-548}$ is obtained, whereas co-expression with *svp* yields holo-form CmnI/CmnI-T/$CmnI_{21-548}$. A general procedure of gene expression, protein production and purification are described in the previous section. The purified protein was concentrated using Amicon Ultra-15 10,000 NMWL concentrators (Merck) in 100 mM NaCl and 20 mM Tris buffer at pH 8.0 for the following activity assay, binding assay, and BLI analysis, and 300 mM NaCl and 50 mM Tris buffer at pH 8.0 for protein crystallization (CmnI). Protein concentration was determined using the BCA kit (Thermo Scientific) following the standard protocol. The purified protein was stored at −80 ºC.

Activity assay of the CmnA-A$_1$-CmnN complex and the CmnA-A$_2$-CmnN complex

The A domain activity assay was determined by a colorimetric pyrophosphate assay.[2] The CmnA-A$_1$-CmnN complex and the CmnA-A$_2$-CmnN complex reactions were carried out in a final volume of 100 μL containing 1mM substrate (L-Dap, L-Ser, L-Cys, L-Ala, or L-Lys). 1 mM $MgCl_2$, 1 mM ATP, 10 mM hydroxylamine (NH_2OH), and 10 μM the purified CmnA-A$_1$-CmnN complex in 20 mM Tris buffer at pH 8.0. The reaction was incubated at 30 °C for 1 h. The reaction mixture was then added sequentially with 1 mL Na_2MoO_4 solution (20 mM Tris/HCl, 500 mM Na_2MoO_4, and 60% v/v ACN) for 3 min and 40 μL bis(triphenylphosphoranylidene) ammonium chloride (50 mM in ACN). The reaction mixture was centrifuged at 18,000 g for 30 min. The pellet was redissolved in 1 mL ACN. After centrifugation at 18,000 g for 10 min, 200 μL supernatant was added with 20 μL ascorbic acid buffer (0.5 mM ascorbic acid, 2 M HCl, and 60 % v/v ACN) for 40 min. The reactions were performed in 96-well plates and the 620 nm absorbance were detected on an EPOCH microplate reader (BioTek).

Mass spectrometric analysis of apo-form CmnI/CmnI-T, holo-form CmnI/CmnI-T, and CmnI/CmnI-T loaded with L-Dap

The apo-form CmnI/CmnI-T was purified from the *E. coli* constructs containing the plasmid pET28a-CmnI/pET28a-CmnI-T, respectively. The holo-form CmnI/CmnI-T was purified from the *E. coli* constructs containing the plasmid pET28a-CmnI/pET28a-CmnI-T with the additional plasmid pCDFDuet-Svp. For CmnI loaded with L-Dap, pET28a-CmnI, pCDFDuet-Svp, and pACYCDuet-CmnA-A$_1$-CmnN (without His-tag) were used to transform *E. coli*. During gene expression and protein production, the LB medium was supplemented with 1 mM of L-Dap. Afterward, CmnI was purified and rapidly analyzed by intact protein mass spectrometry. For CmnI-T loaded with L-Dap, the holo-form CmnI-T and CmnA-A$_1$-CmnN complex were purified individually and then mixed together. 1 mM ATP and Mg^{2+} were added to the enzyme reaction. After 1hr, the reaction was subjected to intact protein mass analysis. Intact protein mass analysis was performed by the Center for Advanced Instrumentation and Department of Applied Chemistry at National Yang Ming Chiao Tung University, Hsinchu, Taiwan. Mass spectra of the various CmnI/CmnI-T protein solutions were acquired by direct infusion (2 μL). ESI(+)MS experiments were carried out using an Impact HD Q-TOF mass spectrometer (Bruker, Germany) equipped with an electrospray ionization (ESI) source operating in positive ion mode. The parameters of ESI(+) included 4.5kV for ion spray voltage, 200 °C for capillary temperature, and 6 L/min for sheath gas flow rate. The mass spectra were collected over the mass range of m/z 50-1500 at

a resolving power of 40000. The collected data were analyzed using Compass DataAnalysis 4.1 (Bruker, Germany).

Determination of the size of CmnI by size-exclusion chromatography

Size-exclusion chromatography for CmnI was performed using a HiLoad Superdex 16/600 column 200 pg (Cytiva) with an NGC Chromatography Systems (Bio-Rad) at 4 °C. 1 mL of sample was loaded per run. The column was calibrated with Gel Filtration Standard (Bio-Rad) and developed with the elution buffer (100 mM NaCl and 20 mM Tris, pH 8.0) at a flow rate of 1 mL/min.

Bio-layer interferometry (BLI) binding kinetics assay

The binding affinity of the CmnA-A_1-CmnN complex and the CmnA-A_2-CmnN complex to holo-form CmnI were measured using the Octet HTX system (FrotéBio) at the Center for Emergent Functional Matter Science, National Yang Ming Chiao Tung University, Taiwan. BLI measurements were carried out at a shaking speed of 1,000 rpm. The purified CmnA-A_1-CmnN complex and the CmnA-A_2-CmnN complex at various concentrations of 2, 4, 8, and 16 μM were prepared in kinetic buffer (500 mM NaCl, 20 mM Tris buffer, pH 8.0, and 10 % v/v, glycerol) and loaded onto Ni-NTA biosensors immobilized by CmnI or $CmnI_{21\text{-}548}$ (Molecular Devices, FortéBio). Four concentrations of the CmnA-A_1-CmnN complex and the CmnA-A_2-CmnN complex in kinetic buffer were added to a black polypropylene 96-well microplate (Greiner Bio-one), with one row containing kinetic buffer as a reference control. Each protein concentration underwent an assay cycle using Ni-NTA-CmnI probes. One assay cycle consists of 60 s of baseline normalization in kinetics buffer, 400 s of association in the protein solution, 200 s of dissociation in kinetics buffer. BLI results were analyzed using FortéBio Data Analysis High Throughput 12.0.

Crystallization and data collection of CmnI

CmnI was crystallized using the hanging drop vapor-diffusion method. CmnI was concentrated to 22 mg/mL and was crystallized in a screen condition, 20% w/v PEG 1000 and 100 mM Tris buffer, pH 8.5 at 5 °C. The protein crystals were transferred to a cryoprotectant solution containing 20% glycerol prior to the X-ray diffraction experiment. The diffraction data of CmnI was collected at National Synchrotron Radiation Research Center (NSRRC), Taiwan, on the 15A1 beamline using a wavelength of 1 Å with the Rayonix MX300HE CCD Area detector. Data were indexed and scaled with HKL2000.

Structure determination and refinement of CmnI

The structure of CmnI was solved by the molecular replacement method of MOLREP[3] using the structure of the T-C didomain NRPS from fuscachelin biosynthesis (PDB entry 7KVW) as a search model.[4] Extensive manual model building was performed using COOT.[5] The models were refined with REFMAC.[6] The atomic coordinate and structure factor of CmnI have been deposited in the Protein Data Bank (PDB) with the accession code 9UAY. Data processing and refinement statistics are summarized in Table S1.

AlphaFold structure prediction

The structure of CmnI-T (Met1−Gly100) was predicted using AlphaFold3 with default parameters on the AlphaFold3 server.[7] Five structural models were generated, and the resulting predictions were presented using the PyMOL Molecular Graphics System (version 2.5.2).

Sequence similarity network (SSN) of CmnI

Over 1,000 homologues of CmnI, ranging from 30% to 100% amino acid sequence identities, were found using BLASTP analysis with the NCBI GenBank non-redundant protein sequence database. 987 collection of the CmnI homologues for SSN analysis was achieved using the Enzyme Function Initiative (EFI) Enzyme Similarity Tool (EST) with CmnI as the query sequence for searching homologues with amino acid sequence identities greater than 35% from the UniProt database and was applied at an e value threshold of 10^{-80}. The SSN was generated using EFI-EST and visualized in Cytoscape 3.9.1.[8] In addition to CmnI and VioI, 11 CmnI homologues were further analyzed using EFI-GNT to identify their potential associated natural product biosynthetic gene clusters.[9]

Supplementary Table

Table S1. Data collection, phasing, and refinement statistics for CmnI structure

	CmnI
**Data collection**	
Space group	C2
Cell dimensions	
a, *b*, *c* (Å)	167.888, 129.520, 61.313
α, β, γ (°)	90.00, 95.15, 90.00
Resolution (Å)	27.65–2.17 (2.25–2.17)
R_{sym} or R_{merge}	0.056 (0.592)
$I / \sigma I$	20.8 (2.2)
Completeness (%)	98.2 (97.5)
Redundancy	3.2 (3.2)
**Refinement**	
Resolution (Å)	27.65–2.17
No. reflections	61605
R_{work} / R_{free}	0.215/0.246
No. atoms	
Protein	6741
Water	130
***B*-factors**	
Protein	38.8
Water	34.2
R.m.s. deviations	
Bond lengths (Å)	0.0057
Bond angles (°)	1.29

Supplementary Figures

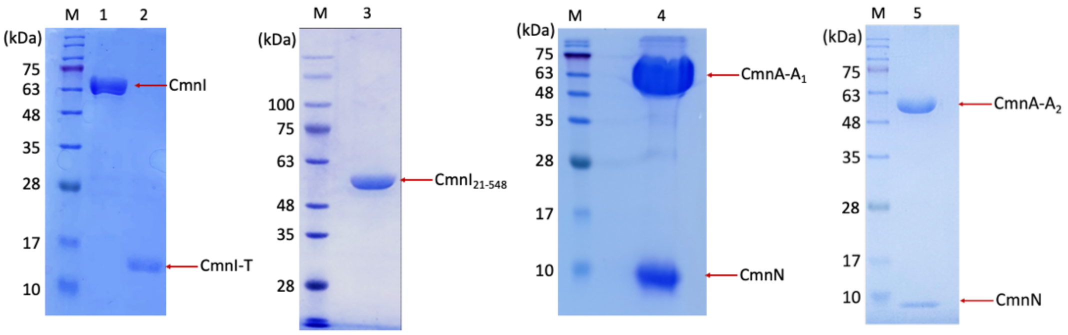

Figure S1. SDS-PAGE and molecular weight estimation of CmnI, CmnI-T, $CmnI_{21-548}$, the $CmnA-A_2$-CmnN complex, and the $CmnA-A_1$-CmnN complex. The calculated molecular weight: CmnI (lane 1), 61.5 kDa; CmnI-T (lane 2), 12.6 kDa; $CmnI_{21-548}$ (lane 3), 59.7 kDa; $CmnA-A_1$ (the band above in lane 4), 56.3 kDa; CmnN (the band below in lane 4), 7.9 kDa; $CmnA-A_2$ (the band above in lane 5), 55.5 kDa.

(a)

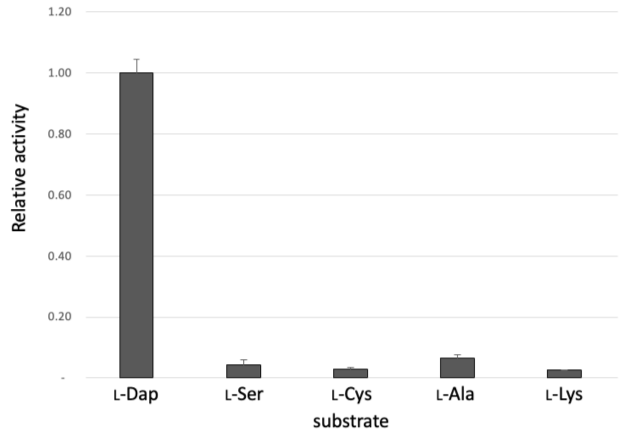

(b)

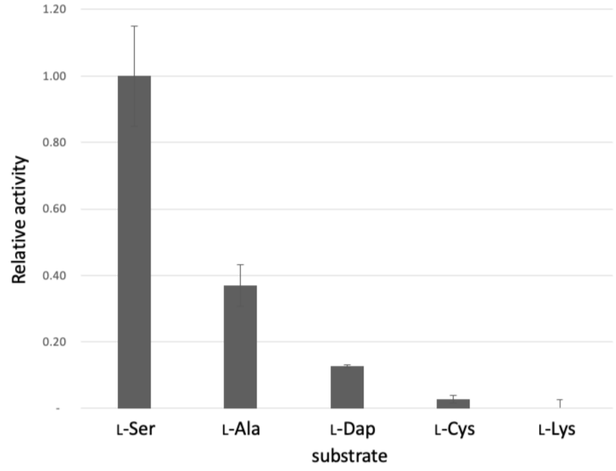

Figure S2. Substrate selectivity of CmnA-A_1 and CmnA-A_2. (a) CmnA-A_1 and cmnN were co-expressed in *E. coli* to form the CmnA-A_1-CmnN complex for A domain activity assay. (b) CmnA-A_2 and cmnN were co-expressed in *E. coli* to form the CmnA-A_2-CmnN complex for A domain activity assay. Each of the activity assays was performed in triplicate (n=3) and the data were presented as mean value +/- standard error of the mean (SEM). Gene cloning, protein production and purification, and enzyme activity assay are described in the Supplementary Methods.

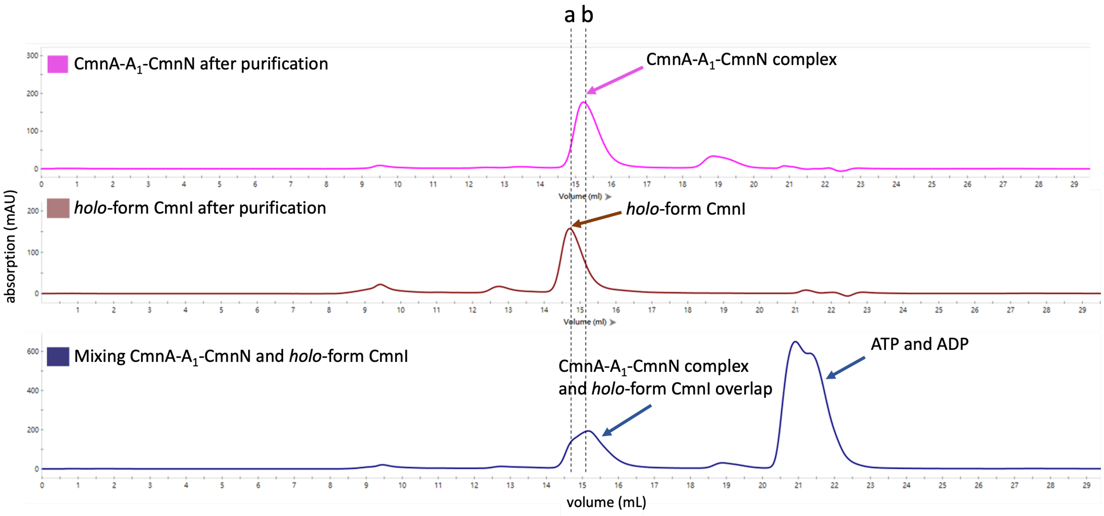

Figure S3. Size-exclusion chromatography analysis of interaction between CmnA-A_1 and CmnI. Size-exclusion chromatography was performed using a Superdex^{TM} 200 Increase 10/300 GL column (Cytiva) with an NGC Chromatography Systems (Bio-Rad) at 4 °C. 0.5 mL of sample was loaded per run. a and b represent the holo-form CmnI and CmnA-A_1-CmnN complex, respectively. When the holo-form CmnI and CmnA-A_1-CmnN complex were mixed and incubated with ATP and L-Dap, no protein complex with a relatively higher molecular weight was observed, suggesting that the holo-form CmnI and CmnA-A_1-CmnN complex do not strongly interact.

(a)

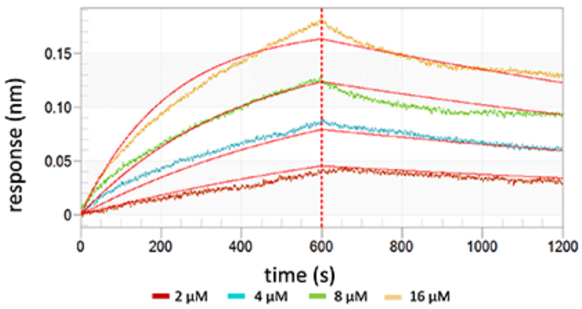

	K_D (M)	K_D Error	K_{on} (1/Ms)	K_{on} Error	K_{off} (1/s)	K_{off} Error	R^2
CmnA-A_1-CmnN	1.85 x10^{-6}	2.61x10^{-8}	258	1.63	4.77x10^{-4}	6.02x10^{-6}	1

(b)

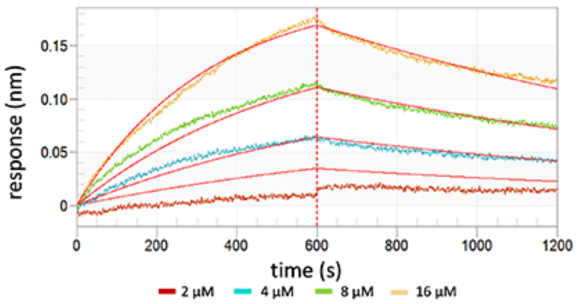

	K_D (M)	K_D Error	K_{on}(1/Ms)	K_{on} Error	K_{off} (1/s)	K_{off} Error	R^2
CmnA-A_2-CmnN	4.93 x10^{-6}	1.06x10^{-7}	148	2.32	7.28x10^{-4}	1.08x10^{-5}	0.96

(c)

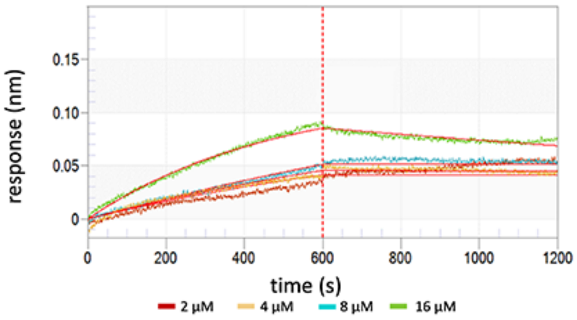

	K_D (M)	K_D Error	K_{on} (1/Ms)	K_{on} Error	K_{off} (1/s)	K_{off} Error	R^2
CmnA-A_1-CmnN	x	x	x	x	x	x	x

Figure S4. Kinetic binding curves of the CmnA-A_1-CmnN complex and the CmnA-A_2-CmnN complex variants interacting with the holo-form CmnI or $CmnI_{21\text{-}548}$. Binding curves show the interaction of holo-form CmnI with (a) the CmnA-A_1-CmnN complex and (b) the CmnA-A_2-CmnN complex, and (c) holo-form $CmnI_{21\text{-}548}$ with the CmnA-A_1-CmnN complex. The x- and y-axis represent the reaction time and BLI signal. The binding curves at four protein concentrations, 2, 4, 8, and 16 µM, are shown in different colors below each figure. The K_D, K_{on}, and K_{off} values are provided in the table.

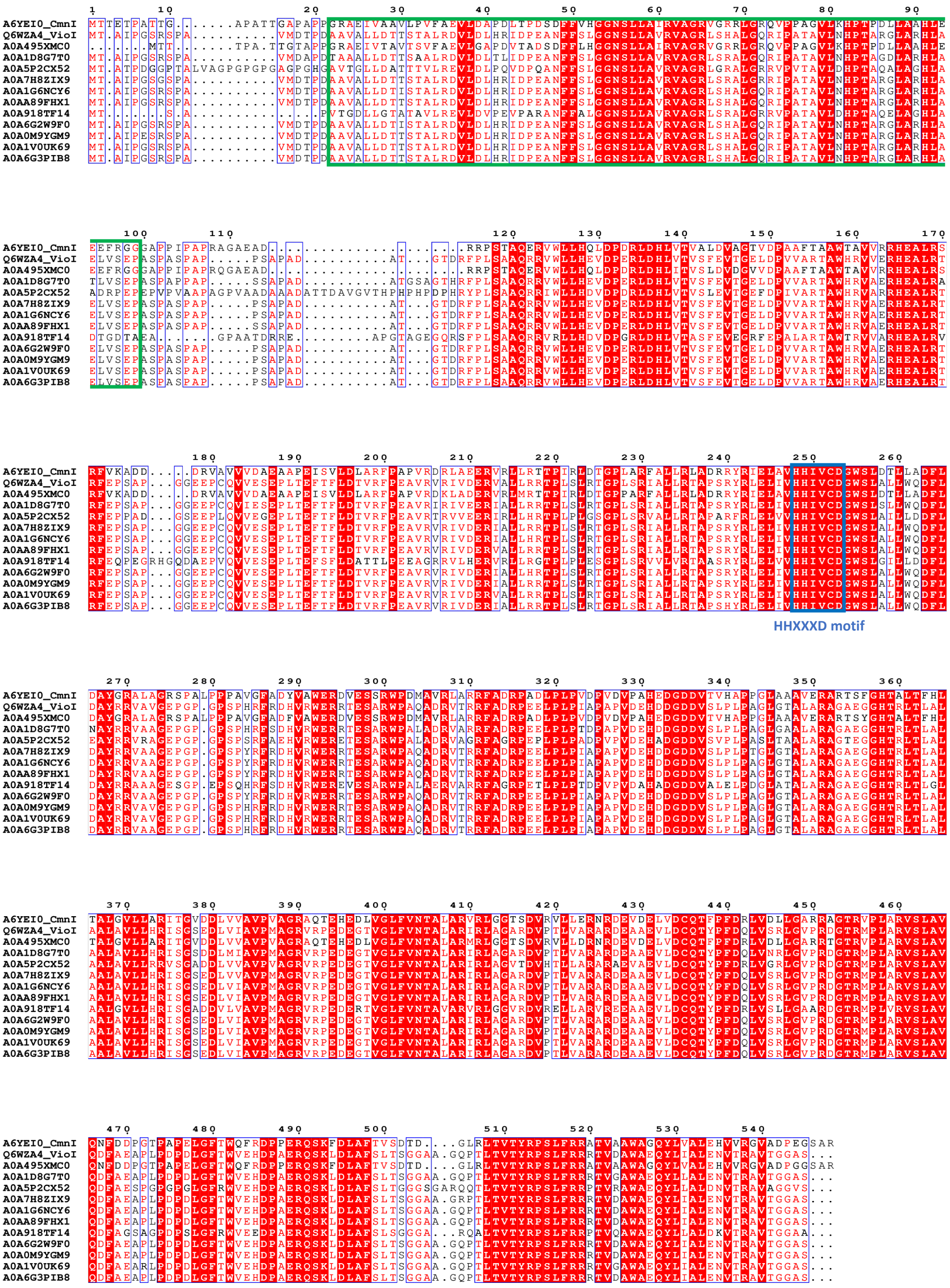

Figure S5. Sequence alignment of CmnI with 12 homologues from the specific cluster. Aligned residues are colored on the bases of the level of conservation (red background shows identity, red character for similarity, and blue frame for similarity across group). The T domain region was highlighted with a green box, based on the crystal structure of the T domain from TycC5-6 (PDB ID: 2JGP).[10] The conserved HHXXXD motif was highlighted with a blue box. The UniProt protein IDs are listed on the left.

A6YEI0_CmnI	100	53.66	94.81	54.22	55.8	54.03	53.28	53.66	56.24	53.28	53.28	53.47	53.28
Q6WZA4_VioI	53.66	100	52.94	94	76.18	98.18	98.91	99.27	70.7	98.91	99.45	99.45	99.27
A0A495XMC0	94.81	52.94	100	53.13	55.04	53.32	52.56	52.94	56.05	52.56	52.56	52.75	52.56
A0A1D8G7T0	54.22	94	53.13	100	76.85	93.64	94	94.36	71.05	94	94.18	94.18	94.36
A0A5P2CX52	55.8	76.18	55.04	76.85	100	77.09	76.73	76.55	72.49	76.73	76.55	76.18	76.55
A0A7H8ZIX9	54.03	98.18	53.32	93.64	77.09	100	98.55	98.55	71.08	98.55	98.36	98	98.55
A0A1G6NCY6	53.28	98.91	52.56	94	76.73	98.55	100	99.27	70.13	100	99.09	98.73	99.27
A0AA89FHX1	53.66	99.27	52.94	94.36	76.55	98.55	99.27	100	70.51	99.27	99.09	99.09	99.27
A0A918TF14	56.24	70.7	56.05	71.05	72.49	71.08	70.13	70.51	100	70.13	70.32	70.51	70.32
A0A6G2W9F0	53.28	98.91	52.56	94	76.73	98.55	100	99.27	70.13	100	99.09	98.73	99.27
A0A0M9YGM9	53.28	99.45	52.56	94.18	76.55	98.36	99.09	99.09	70.32	99.09	100	99.27	99.45
A0A1V0UK69	53.47	99.45	52.75	94.18	76.18	98	98.73	99.09	70.51	98.73	99.27	100	99.09
A0A6G3PIB8	53.28	99.27	52.56	94.36	76.55	98.55	99.27	99.27	70.32	99.27	99.45	99.09	100

Figure S6. Sequence alignment identity matrix of CmnI with 12 homologues from the specific cluster. The UniProt protein IDs are listed on the left.

(a)

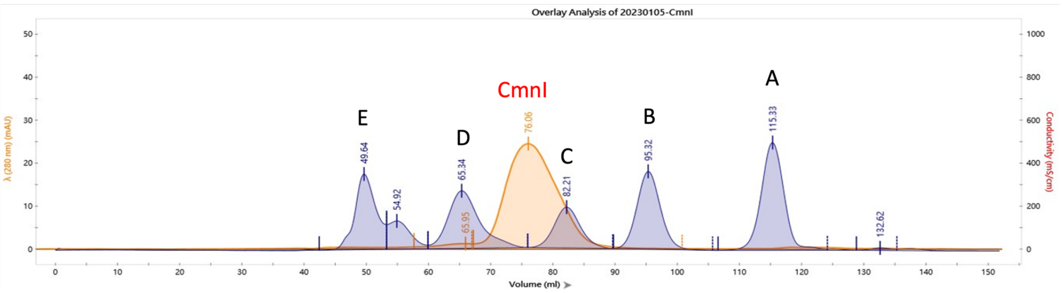

(b)

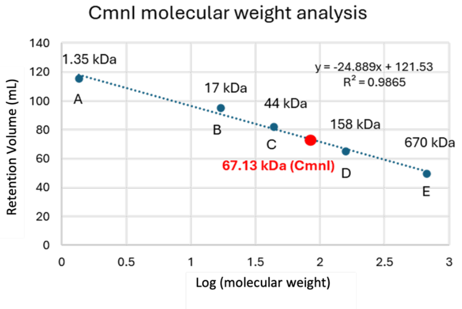

Figure S7. Molecular weight estimation of CmnI. (a) Size-exclusion chromatography for CmnI was performed using a HiLoad Superdex 16/600 column 200 pg (Cytiva) with an NGC Chromatography Systems (Bio-Rad). The running condition is described in the Supplementary Methods. (b) Molecular weight estimation of CmnI by size exclusion chromatography. The apparent molecule weights of CmnI is estimated to be 67.13 kDa, suggesting that CmnI (calculated molecular weight of 61.48 kDa) exists as a monomer.

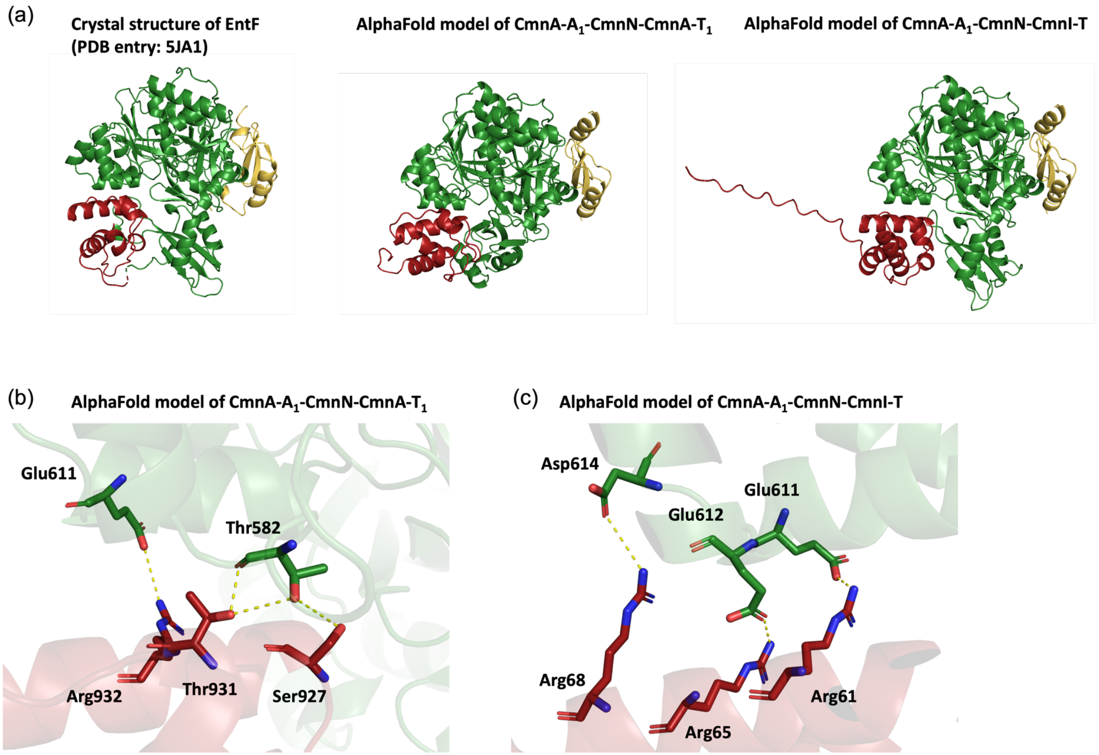

Figure S8. Potential interactions between CmnA-A$_1$ and the two T domains. (a) The crystal structure of EntF (PDB entry: 5JA1) and the AlphaFold complex models of CmnA-A$_1$-CmnN-CmnA-T$_1$ and CmnA-A$_1$-CmnN-CmnI-T. The A domain, T domain, and MbtH-like protein are colored green, red, and yellow, respectively. (b) Local view of the potential interactions between CmnA-A$_1$ and CmnA-T$_1$. (c) Local view of the potential interactions between CmnA-A$_1$ and CmnI-T. Hydrogen bonds and electrostatic interactions are shown as yellow dotted lines.

Supplementary References

(1) Sanchez, C.; Du, L.; Edwards, D. J.; Toney, M. D.; Shen, B. Cloning and characterization of a phosphopantetheinyl transferase from Streptomyces verticillus ATCC15003, the producer of the hybrid peptide-polyketide antitumor drug bleomycin. *Chem. Biol.* **2001**, *8*, 725−738.

(2) Katano, H.; Watanabe, H.; Takakuwa, M.; Maruyama, C.; Hamano, Y. Colorimetric determination of pyrophosphate anion and its application to adenylation enzyme assay. *Anal. Sci.* **2013**, *29*, 1095−1098.

(3) Vagin, A.; Teplyakov, A. Molecular replacement with MOLREP. *Acta Crystallogr. D Biol. Crystallogr.* **2010**, *66*, 22−25.

(4) Izore, T.; Candace Ho, Y. T.; Kaczmarski, J. A.; Gavriilidou, A.; Chow, K. H.; Steer, D. L.; Goode, R. J. A.; Schittenhelm, R. B.; Tailhades, J.; Tosin, M.; et al. Structures of a non-ribosomal peptide synthetase condensation domain suggest the basis of substrate selectivity. *Nat. Commun.* **2021**, *12*, 2511.

(5) Emsley, P.; Lohkamp, B.; Scott, W. G.; Cowtan, K. Features and development of Coot. *Acta Crystallogr. D Biol. Crystallogr.* **2010**, *66*, 486−501.

(6) Winn, M. D.; Murshudov, G. N.; Papiz, M. Z. Macromolecular TLS refinement in REFMAC at moderate resolutions. *Methods Enzymol.* **2003**, *374*, 300−321.

(7) Jumper, J.; Evans, R.; Pritzel, A.; Green, T.; Figurnov, M.; Ronneberger, O.; Tunyasuvunakool, K.; Bates, R.; Zidek, A.; Potapenko, A.; et al. Highly accurate protein structure prediction with AlphaFold. *Nature* **2021**, *596*, 583−589.

(8) Zallot, R.; Oberg, N.; Gerlt, J. A. The EFI web resource for genomic enzymology tools: Leveraging protein, genome, and metagenome databases to discover novel enzymes and metabolic pathways. *Biochemistry* **2019**, *58*, 4169−4182.

(9) Oberg, N.; Zallot, R.; Gerlt, J. A. EFI-EST, EFI-GNT, and EFI-CGFP: Enzyme Function Initiative (EFI) web resource for genomic enzymology tools. *J. Mol. Biol.* **2023**, *435*, 168018.

(10) Samel, S. S.; Schoenafinger, G.; Knappe, T. A.; Marahiel, M. A.; Essen, L. -O. Structural and functional insights into a peptide bond-forming bidomain form a nonribosomal peptide synthetase. *Structure* **2007**, *15*, 728−792
